# Molecular Subclassification Based on Crosstalk Analysis Improves Prediction of Prognosis in Colorectal Cancer

**DOI:** 10.3389/fgene.2021.689676

**Published:** 2021-11-04

**Authors:** Xiaohua Liu, Lili Su, Jingcong Li, Guoping Ou

**Affiliations:** ^1^ State Key Laboratory of Oncology in South China, Sun Yat-sen University Cancer Center, Guangzhou, China; ^2^ School of Electronic and Information Engineering, Xi’an Jiaotong University, Xi’an, China

**Keywords:** colorectal cancer, pathways deregulation score, overall survival, signature, personalized medicine

## Abstract

The poor performance of single-gene lists for prognostic predictions in independent cohorts has limited their clinical use. Here, we employed a pathway-based approach using embedded biological features to identify reproducible prognostic markers as an alternative. We used pathway activity score, sure independence screening, and K-means clustering analyses to identify and cluster colorectal cancer patients into two distinct subgroups, G2 (aggressive) and G1 (moderate). The differences between these two groups with respect to survival, somatic mutation, pathway activity, and tumor-infiltration by immunocytes were compared. These comparisons revealed that the survival rates in the G2 subgroup were significantly reduced compared to that in the G1 subgroup; further, the mutational burden rates in several oncogenes, including *KRAS*, *DCLK1*, and *EPHA5*, were significantly higher in the G2 subgroup than in the G1 subgroup. The enhanced activity of the critical pathways such as MYC and epithelial-mesenchymal transition may also lead to the progression of colorectal cancer. Taken together, we established a novel prognostic classification system that offers meritorious insights into the hallmarks of colorectal cancer.

## Introduction

Colorectal cancer is the third most common form of cancer and the second leading cause of cancer-related deaths worldwide (Bray et al., 2018). Currently, the TNM staging system is widely used to predict the prognosis of colorectal cancer patients; however, even patients within the same TNM stage often present with distinct prognosis outcomes in clinical practice. For instance, patients with stage I and II colorectal cancer generally exhibit a favorable prognosis and are treated with surgical resection alone. Unfortunately, approximately 10–30% of stage I and II colorectal cancer patients experience tumor recurrence within 5 years of curative surgery and require more intense treatment, such as adjuvant chemotherapy (Lin et al., 2017; Guraya, 2019). Therefore, more precise prognostic tools for colorectal cancer will enable individualized therapy and improve patient prognosis.

Bioinformatic subtyping methods are generally based on gene expression data. There are two examples of these systems for colorectal cancer, where subtypes are established based on their molecular features, with these subtypes often demonstrating significant differences in clinical outcomes (Luo et al., 2020; Yang et al., 2020). Recently, several research groups have indicated that pathway analysis may be helpful in extracting more stable and interpretable features for risk prediction (Alcaraz et al., 2017; Sheng et al., 2019; Su et al., 2021). Several efforts have been made to decode cancer at the gene, protein, and metabolite levels, and with the help of predefined pathways available from various biological databases, including Kyoto Encyclopedia of Genes and Genomes (KEGG) (Kanehisa et al., 2016), Reactome (Fabregat et al., 2018), and Gene-Set Enrichment Analysis (GSEA) (He et al., 2018a), more stable and interpretable characteristics could be obtained. Usually we calculate the pathway activity difference based the differentially expressed genes calculated based on the comparison of two groups, this method was used to compare the pathway difference between two groups and reveal novel mechanisms, but not suitable for model construction. (Alcaraz et al., 2017). Notably, PARADIGM and Pathifier are exceptions (Vaske et al., 2010; Drier et al., 2013). The Pathifier algorithm only needs the gene expression data from each pathway to produce a coarse-grained score, which represents the degree of dysregulation within related pathways. This means that this algorithm can produce a useful score for evaluating disease subtypes and has been used for subtyping tumors and predicting prognosis in cancer patients (Livshits et al., 2015; Huang et al., 2016; Fa et al., 2019). PARADIGM has been used to infer patient-specific pathway activities from multidimensional cancer genomics data, and could be helpful in integrating multi-omics data and facilitating biomarker discovery in specific diseases (Han et al., 2021; Park et al., 2021). However, since most of the pathways share genes between them, which we call “crosstalk,” the specificity of the pathway activity score (PAS) is compromised. Taking intersection genes among pathways into account on PAS quantification will help identify disease-specific features. Thus, developing new prognostic classifier of colorectal cancer based on crosstalk eliminated PAS would be valuable.

Here we developed a new PAS estimation method based on crosstalk factorization, and established a novel pathway-level-feature-based signature for colorectal cancer that can be used to predict overall survival (OS) outcomes, and serve as a complement to the currently available staging system.

## Material and Methods

### Data Sources

Both mRNA normalized level 3 expression and colorectal cancer clinical data were downloaded from the TCGA portal (https://portal.gdc.cancer.gov/). The microarray data and clinical information from GSE17537, GSE29623, and GSE87211 were downloaded from the GEO database (https://www.ncbi.nlm.nih.gov/geo/). After removing samples without survival data, 613, 55, 65, and 196 samples were retained in these four datasets, respectively.

### Pathway Activity Score

The pathway activity score (PAS) for each dataset was calculated based on the method proposed by Bhandari et al. (2019). We downloaded all pathways from the gene oncology (GO) database (http://geneontology.org/) and generated a new mRNA expression matrix that contains only genes exist in it for each pathway. After that, for each gene, based on its expression level, we classified the tumors into two subgroups, the samples in the higher group were scored +1, while the others were scored −1. Finally, we averaged all gene scores in this pathway as the pathway activity score for each tumor sample. A higher PAS indicates a higher pathway activity in the sample, and otherwise, a lower score means lower activity in the sample.

### Overall Design and Construction of the Prediction Pipeline

**FIGURE 1:**
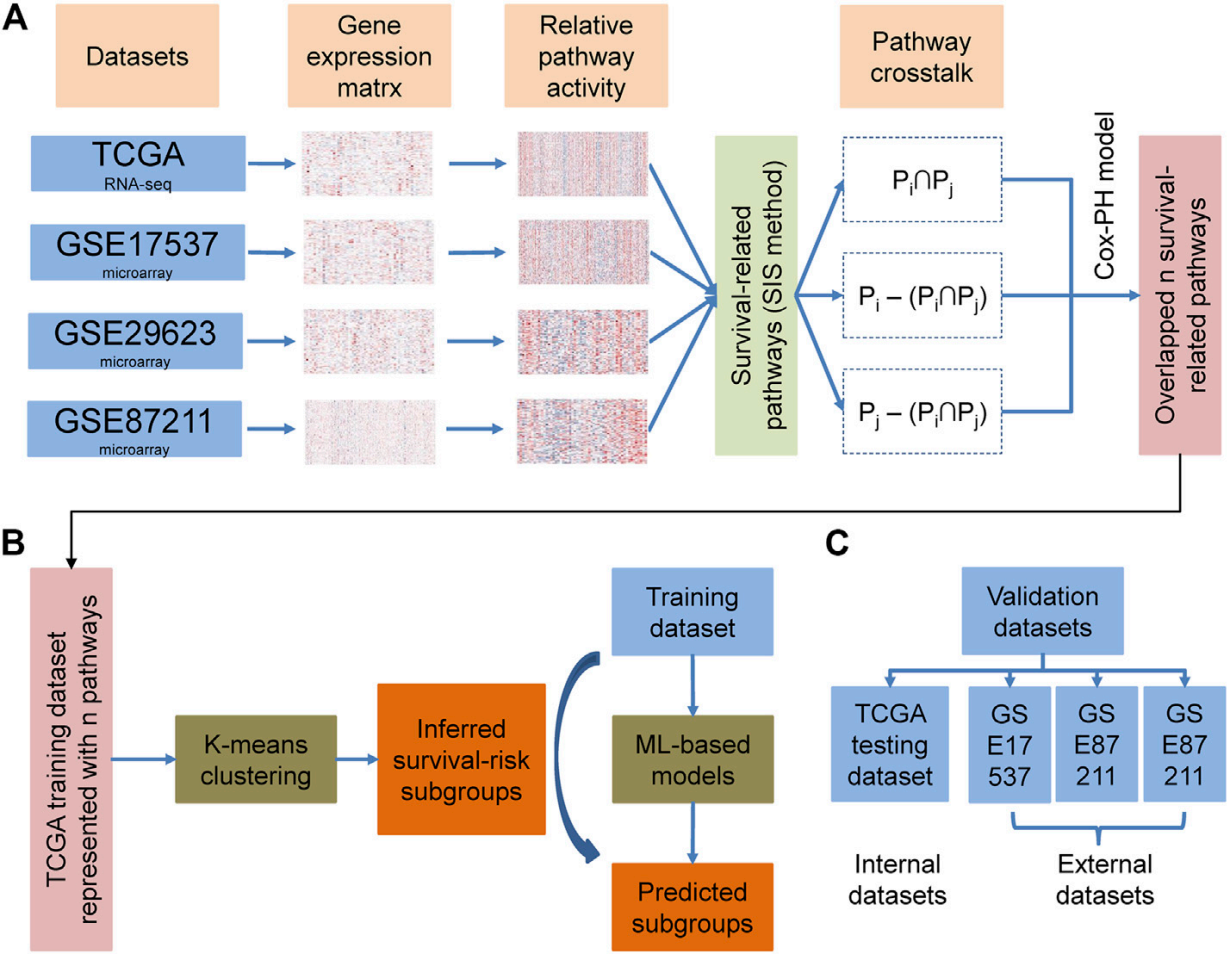
Overall workflow describing the design and validation processes used in this study.

The overall methodology used to define the cancer survival risk subtypes identified in this study is shown in Figure 1. Given a series of data sets, based on PASs and survival information, we calculated the log-rank pvalue for each pathway by regression analysis. The pathways were then ranked based on the log-rank pvalue. Then we applied sure independence screening (SIS) to identify the main pathways associated with overall survival in each cohort at a critical threshold of 100. This value was much larger than the default n/log(n) for each cohort, where n is the sample size. We then used these 100 survival-related pathways (Supplementary Table S1) to evaluate the impact of crosstalk between these pathways on different datasets. The crosstalk between the two pathways can be divided into three categories: $P_{i\cap j}$ , representing the overlapping genes between pathways i and j ; $P_{i}$ – $\left(P_{i\cap j}\right)$ , representing the genes specific to pathway i ; and $P_{j}$ – $\left(P_{i\cap j}\right)$ , representing the genes specific to pathway j . We only retained the crosstalk results where each pathway included at least three genes in the $P_{i\cap j}$ category. We then recalculated the PAS for each sub-pathway and calculated their survival risk p-value using the Cox-PH model. After correction, important (FDR p-value < 0.01) pathways/sub-pathways were determined for each of the three cohorts, and the common pathways/sub-pathways in each data set were used as sample features for further analysis.

We then divided the TCGA data into a training set and a test set using a 4:1 ratio. We then used the activity matrix for each sample to complete a K-means clustering evaluation on the training set and defined the number of clusters as n (2 ≤ *n* ≤6) and then used this evaluation to divide the samples into several subgroups. The optimal number of clusters was determined using three parameters: The C index for the prognostic differences, the Silhouette index, and the Calinski–Harabasz criterion (Supplementary Table S4). This analysis allowed us to classify these samples into two distinct subgroups which were redefined as G1 and G2. We then went on to evaluate these two subgroups in other datasets, using various machine-learning frameworks, such as SVM, Adaboost, knearest neighbor (KNN), and Gaussian, and used the pathway activity matrix to build a novel classification model. These algorithms were applied to our frameworks via the Python package “sklearn,” which integrated many classification and regression algorithms, using default parameters. We were able to use a comparison of these predictions to produce an optimal algorithm which was then applied to our final model. We finally settled on the KNN classification algorithm and this was used for further implementation (Supplementary Table S2). The number of neighbors was set to 5, the leaf size passed to the classification tree was set to 30, and the power parameter for the Minkowski metric was set to 2.

### Evaluation Metrics for Model

We trained our novel classification model using 10-fold cross-validation of the TCGA training dataset and used this to determine the best machine learning framework, as described above. We then went on to evaluate the performance of this classification method using the TCGA test dataset as well as the three GEO datasets. We combined the C-index, Brier score, and log-rank p-value with a deep learning-based study to reflect the prediction accuracy of these methods. The application of these metrics allowed us to quantify the proportion of patient pairs whose prediction was consistent with their OS outcome (Harrell et al., 1996). The C-index was determined using the R “survcomp” package (Schröder et al., 2011), and the Brier score was used to measure the accuracy of the probabilistic predictions. Kaplan–Meier and log-rank analyses were used to compare the survival differences between the groups (R survival package available from http://CRAN.R-project.org/package=survival) and the mean differences between the predicted and observed survival rates at specific time points were determined using the survival analyses metrics, and this score was negatively associated with accuracy and determined using the R “survcomp” package.

### Differentially Expressed Genes and GSEA Analyses

Differentially expressed genes between the G2 and G1 subgroups were identified using the DESeq2 package (Love et al., 2014) and GSEA analyses were used to compare the differences between these two subgroups at the hallmark pathway level (Mootha et al., 2003; Subramanian et al., 2005).

### Clinical Covariate and Somatic Mutation Analyses

We compared the somatic mutation rates between the G2 and G1 subgroups in the TCGA training cohort using Fisher’s exact test and compared the distributions of various clinicopathological features, such as tumor stage, new tumor event, and sex in each of these subgroups using Fisher exact tests.

## Results

### Identification of Prognostic Subtypes in Colorectal Cancer

The overall methodology used to identify the different cancer survival risk subtypes defined in this study is shown in Figure 1. We produced four curated colorectal cancer datasets (TCGA, GSE17537, GSE29623, and GSE87211) using the survival information from the TCGA and GEO databases (Table 1). We then used the gene expression matrix from these datasets to calculated the PAS for each pathway identified using the KEGG and Gene Ontology Resource databases and then extracted the pathways most closely associated with overall survival. These were then evaluated using the SIS method to identify the central pathways associated with overall survival when using a critical threshold of 100 (Supplementary Table S1). We then explored the impact of crosstalk between these pathways on each of the datasets and selected the most important sub-pathways (FDR p-value < 0.01) for each.

**TABLE 1:** Clinicopathological features for each of the enrolled cohorts.

Parameters		TCGA training (*n* = 510)	TCGA testing (*n* = 128)	GSE17537 (*n* = 55)	GSE29623 (*n* = 65)	GSE87211 (*n* = 196)
Age	<60	152 (29.8%)	31 (24.22%)	24 (43.64%)	0 (0%)	73 (37.24%)
	≥60	358 (70.2%)	97 (75.78%)	31 (56.36%)	0 (0%)	123 (62.76%)
	Not reported	0 (0%)	0 (0%)	0 (0%)	65 (100%)	0 (0%)
Gender	Male	272 (53.33%)	65 (50.78%)	26 (47.27%)	40 (61.54%)	136 (69.39%)
	Female	237 (46.47%)	61 (47.66%)	29 (52.73%)	25 (38.46%)	60 (30.61%)
	Not reported	1 (0.2%)	2 (1.56%)	0 (0%)	0 (0%)	0 (0%)
OS	Alive	386 (75.69%)	101 (78.91%)	35 (63.64%)	40 (61.54%)	168 (85.71%)
	Dead	104 (20.39%)	22 (17.19%)	20 (36.36%)	25 (38.46%)	28 (14.29%)
	Not reported	20 (3.92%)	5 (3.91%)	0 (0%)	0 (0%)	0 (0%)

OS, overall survival; TCGA, the Cancer Genome Atlas; *n*, number.

**FIGURE 2:**
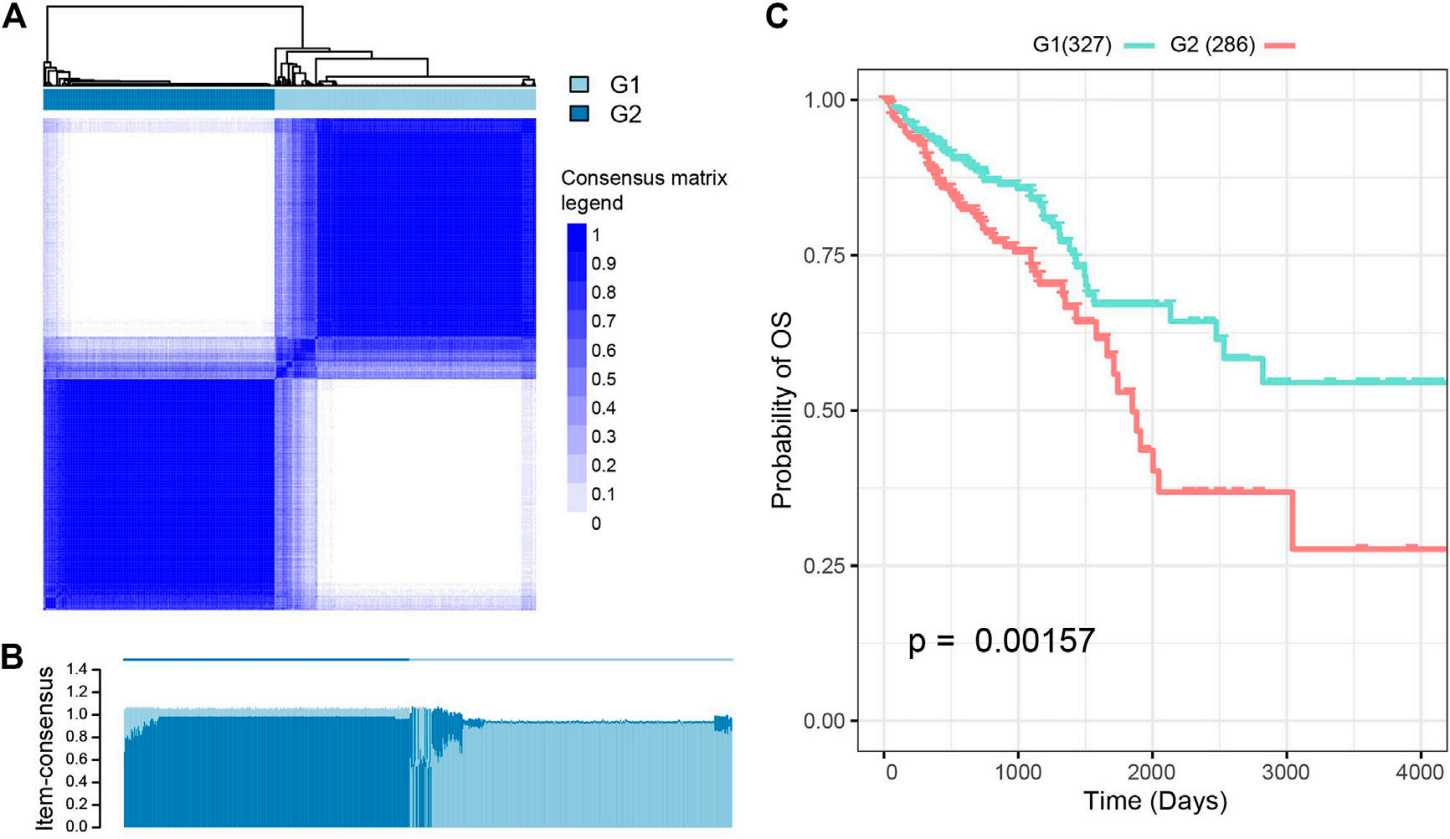
Subtyping analysis of the Cancer Genome Atlas-colorectal cancer (TCGA-CRC) patients. **(A)** K-means clustering analysis split these patients into two subgroups. The optimal number of clusters was determined using three parameters: The C index for prognostic differences, the Silhouette index, and the Calinski–Harabasz criterion. **(B)** Performance of K-means clustering when k was set at 2. **(C)** There were significant differences in survival rates between these two CRC subtypes.

These analyses identified 11 central features (Supplementary Table S3) which were then used to perform a K-means clustering analysis which produced two subgroups: G1 and G2 (Figures 2A,B; Supplementary Table S4). Patients from the G2 subgroup displayed significantly worse overall survival than those in the G1 subgroup (Figure 2C; *p* = 0.0015, log-rank test).

### Evaluating the Performance of Pathway-Based Prognosis

**FIGURE 3:**
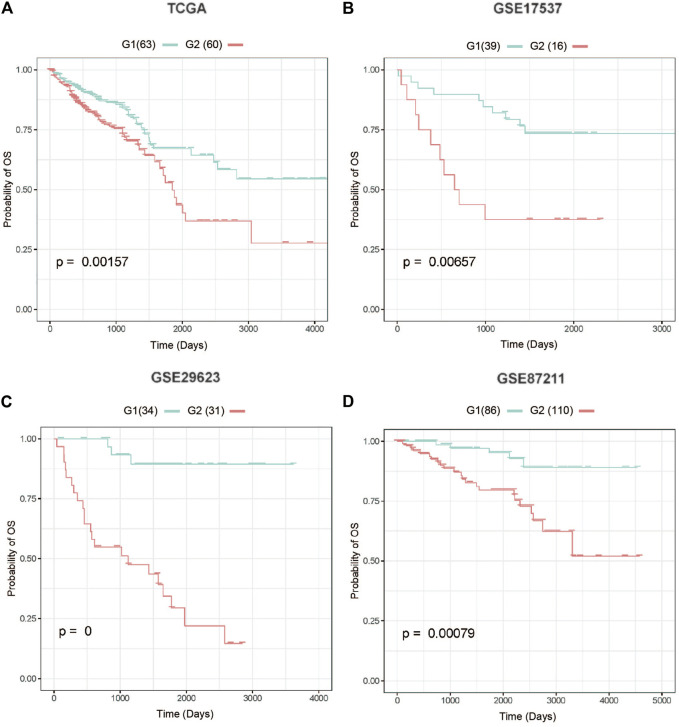
Four test cohorts demonstrating significant differences in survival rates. **(A)** The Cancer Genome Atlas (TCGA) test cohort, **(B)** GSE17537 cohort, **(C)** GSE29623 cohort, **(D)** GSE87211 cohort. G2: aggressive (higher-risk survival) subtype; G1: moderate (lower-risk survival) subtype.

The AUC values for the training and test datasets were 0.9305 and 0.8909, respectively, and the ROC curves are shown in Supplementary Figure S1. In addition, we observed a considerable performance in prediction accuracy when using the PAS features as evidenced by the C-index value and significant differences in OS between G1 and G2, as evidenced by the log-rank p-value test (Figure 3; Table 2). We also noted that this model produced outcomes with low Brier error rates. The test data from the TCGA-COADREAD samples produced a high C-index (0.626), low Brier score (0.229), and significant average log-rank p-value (2.90e-3) when evaluated for differences in survival. We then validated this model using three external cohorts (Supplementary Table S5). The log-rank and Kaplan-Meier analyses of the GSE17537, GSE29623, and GSE87211 cohorts produced similar results to the TCGA cohort (log-rank p-value < 0.05, Figure 3; Table 2) indicating that the model was stable. Taken together, these results indicate that this newly developed method can accurately predict OS outcomes in colorectal cancer patients.

**TABLE 2:** Performance of the gene signature in the test TCGA cohort and three external validation cohorts using all 11 features.

Cohort	Omics type	Sample number	C-index	Brier score	Log-rank *P*-value
TCGA	RNA-Seq	123	0.626	0.229	2.90E-03
GSE17537	Microarray	55	0.651	0.227	6.57E-03
GSE29623	Microarray	65	0.747	0.301	3.56E-07
GSE87211	Microarray	196	0.658	0.15	7.91E-04

TCGA, the Cancer Genome Atlas.

### Associations Between Different Survival Subtypes and Genomic Features

**FIGURE 4:**
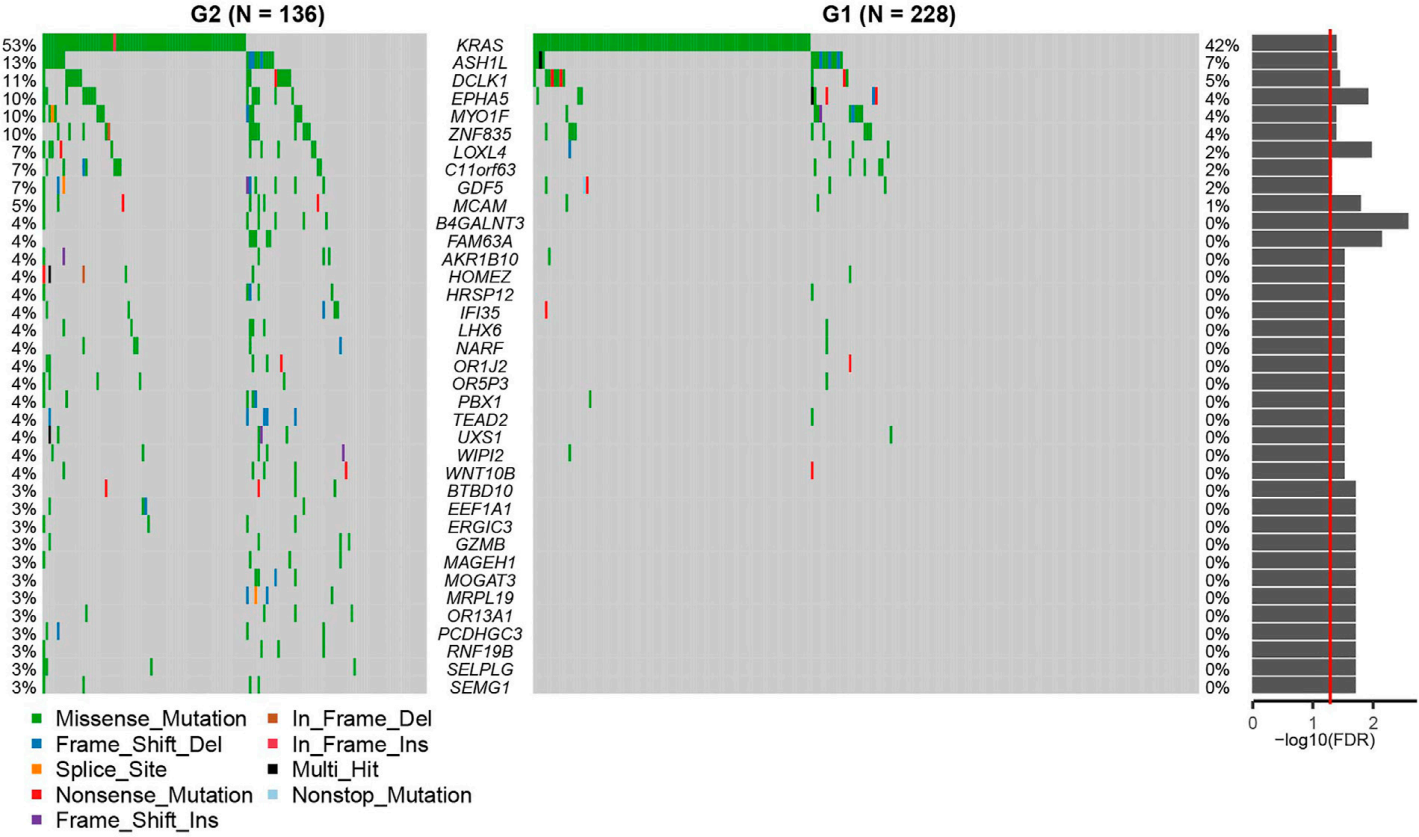
The Oncoprint demonstrating the differences between G2 and G1 subgroups at the genetic level. G2: aggressive (higher-risk survival) subtype; G1: moderate (lower-risk survival) subtype. The p values from the Fisher-exact test are displayed on the right as a bar plot. The red line indicates *p* = 0.05.

**FIGURE 5:**
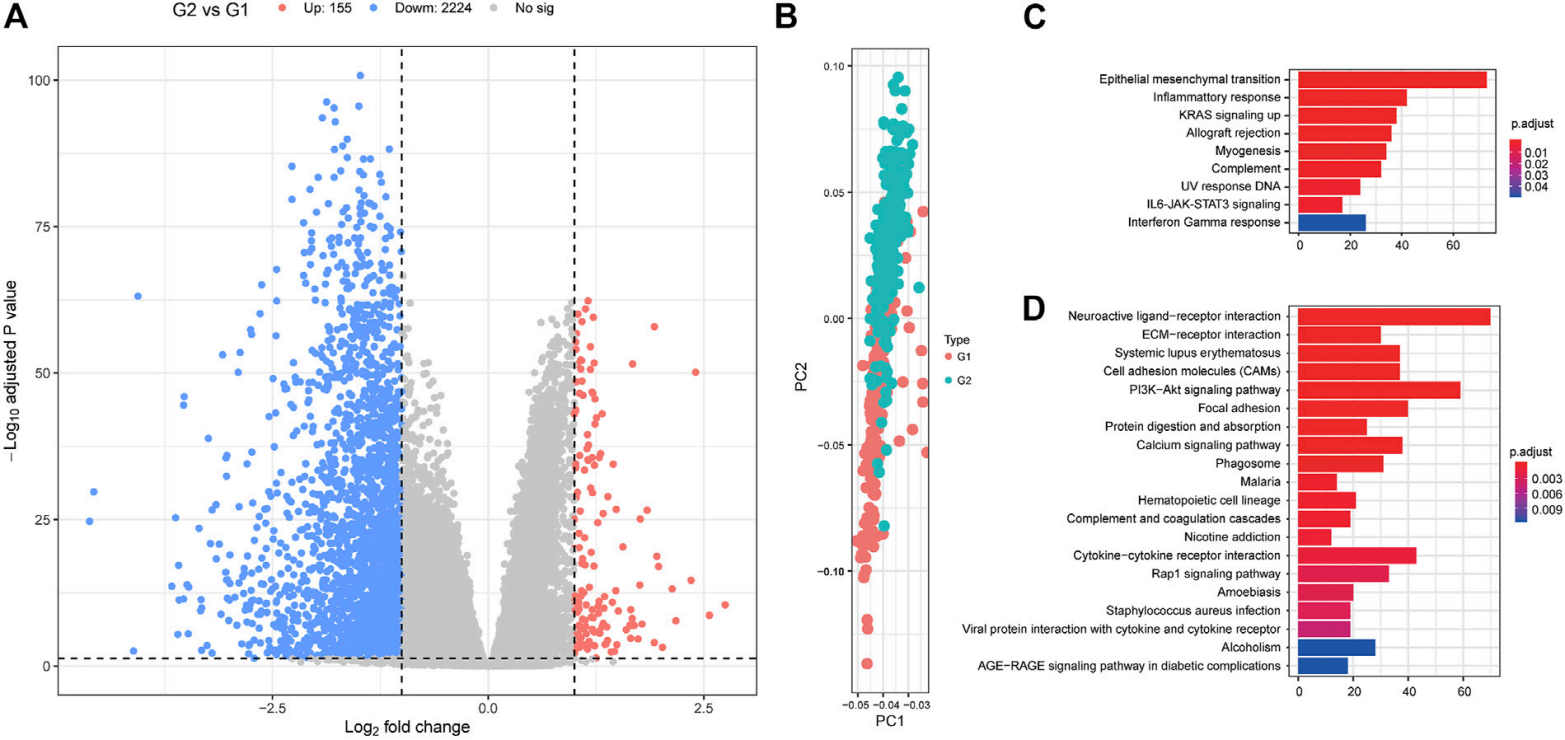
The differentially expressed genes between G1 and G2 subgroups. **(A)** Volcano plot displaying the differentially expressed genes between G2 and G1 subgroups. **(B)** Principal component analysis (PCA) analysis describing the differences in clustering between the G1 and G2 subgroups. Hallmark **(C)** and Kyoto Encyclopedia of Genes and Genomes (KEGG) **(D)** analyses for the differentially expressed genes. G2: aggressive (higher-risk survival) subtype; G1: moderate (lower-risk survival) subtype.

We then used a Fisher’s exact test to evaluate differences between the two survival subtypes identified in the TCGA cohort. These evaluations revealed that KRAS mutations were significantly more frequent in the aggressive subgroup G2 (OR = 1.573, 95%CI: 1.004–2.470, FDR = 0.039, Figure 4; Supplementary Table S6). This was also shown to be true in several other oncogenes including ASH1L, DCLK1, EPHA5, MYO1F, ZNF835, LOXL4, C11orf63, GDF5, MCAM, B4GALNT3, FAM63A, AKR1B10, HOMEZ, HRSP12, IFI35, LHX6, NARF, OR1J2, OR5P3, PBX1, TEAD2, UXS1, WIPI2, WNT10B, BTBD10, EEF1A1, ERGIC3, GZMB, MAGEH1, MOGAT3, MRPL19, OR13A1, PCDHGC3, RNF19B, SELPLG, and SEMG1 (all *p* < 0.05). Differentially expressed genes from each subtype were identified using the DESeq2 package in R and evaluated using a cutoff value of |log_2_ fold change| > 1 and FDR P-value < 0.05 (Love et al., 2014). These analyses identified 155 upregulated and 2,224 downregulated genes in the aggressive G2 subgroup (Figure 5; Supplementary Table S7). Of the upregulated genes, several were found to be associated with survival and pathogenesis in other studies including one study which found that ectopic expression of HBE1 decreased the production of radiation-induced intracellular reactive oxygen species (ROS) and cell mortality (Park et al., 2019); similarly, ORM1 serves as a prognostic factor and can be used to predict therapeutic response in advanced extranodal NK/T cell lymphoma patients treated with pegaspargase/gemcitabine (Zhou et al., 2016). We also tested the correlations between the two survival subtypes (G1 and G2) and the clinicopathological characteristics of the patients in the TCGA cohort and found no significant differences in age, sex, clinical stage or new tumor event subgroups (Supplementary Figure S2).

### The Distribution of Tumor-Infiltrating Immune Cells in G1 and G2 Patients

Differences in the distribution of 22 subpopulations of TIICs in the G1 and G2 subgroups were determined using the CIBERSORT algorithm and the information from the TCGA dataset and the results of this evaluation are summarized in Supplementary Figure S3. We evaluated the average proportion of each immune cell type in both the G1 and G2 subgroups and demonstrated that there were no significant differences in TIIC distribution between these two subgroups. These results suggest that the differences in G1 and G2 survival are not reflected in the TIIC population in these patients.

### Hallmark Analyses Compared the Differences Between G2 and G1 Subgroups

We used GSEA analyses to compare the G2 and G1 subgroups to identify the critical pathways involved in the progression of colorectal cancer. This hallmark pathway enrichment analysis revealed that the differences between these two groups were concentrated in the coagulation, MYC targets v2, epithelial-mesenchymal transition, bile acid metabolism, and peroxisome pathways (FDR p-value < 0.05, Supplementary Figure S4; Supplementary Table S8). This is supported by the fact that most basic research suggests that there is a close connection between hemostatic components and cancer biology as they interact in multiple ways. The coagulation system can be activated by cancer cells, and hemostatic factors can promote tumor progression. In the case of both the MYC (Sikora et al., 1987; Arango et al., 2003; Castell and Larsson, 2015; Boudjadi and Beaulieu, 2016; He et al., 2018b) and EMT (Bates et al., 2007; Vu and Datta, 2017a; Vu and Datta, 2017b; Lamprecht et al., 2018; Ieda et al., 2019; Huang et al., 2020) pathways, many studies have investigated their role in colorectal cancer. We also analyzed the KEGG and Reactome GSEA analyses, and the detailed results are presented in Supplementary Tables S9, S10. These evaluations identified several pathways that are significantly associated with tumor progression and resistance to drug treatment. We also used a GSEA-Reactome analysis to compare the G2 and G1 subgroups, and noted the appearance of several significantly enriched pathways including 3’UTR Mediated Translational Regulation, Bile Acid, and Bile Salt Metabolism, Translation, Cytochrome P450 Arranged by Substrate Type, CDK-Mediated Phosphorylation and Removal of CDC6, P53 dependent G1 DNA damage response, P53 independent G1 S DNA damage checkpoint, S phase, and base excision repair. These results suggest that the oncogenic role of these critical pathways may promote colorectal cancer progression.

## Discussion

Here, we classified colorectal cancer patients into different risk categories based on the results of our pathway activity score with crosstalk evaluations. Our pathway-level features produced satisfactory outcomes in the TCGA training and three external validation cohorts derived from the GEO database. These results were then used to separate the colorectal cancer patients into aggressive (G2) and moderate (G1) subgroups in the TCGA cohort and the other three colorectal cancer datasets (GSE17537, GSE29623, and GSE87211) with reasonable accuracy. We then went on to evaluate these subtype distinctions in terms of other clinicopathological criteria and revealed that while vastly different in terms of survival there were no significant differences in their clinical presentation. However, when we compared the somatic mutation landscape between these two subgroups we found that the mutation rate in oncogene *KRAS* was significantly higher in the G2 group, which may explain why these patients were seen as experiencing a more aggressive progression than the G1 group. Strategies to improve outcomes for patients with *KRAS* mutations should be developed. Taken together our data suggests that evaluating the specific genes governing these important features may provide valuable insights into the hallmarks of colorectal cancer and that these may be combined to produce specific prognostic signatures.

RAS is one of the most investigated proto-oncogenes in the world with gain-of-function mutations in this gene being identified in approximately 30% of all human cancers (Artale et al., 2008; Liu et al., 2019). Furthermore, KRAS mutations are associated with poor prognosis in several cancers, and there is still a lack of effective targeted therapeutics designed to counteract the effects of this mutation. Phipps et al. (2013) enrolled 1,989 colorectal patients in their study which was designed to investigate the association between KRAS mutation and survival, and their results suggested that 31% of these patients had KRAS mutations and that these mutations were closely associated with unfavorable outcomes when compared to the wild type (HR = 1.37, 95%CI: 1.13–1.66). Kim et al. (2016) evaluated the impact of KRAS mutations on time to recurrence (TTR) and overall survival (OS) in patients with metastatic colorectal cancer who underwent curative surgery with perioperative chemotherapy. They found that 37.8% of these patients has a KRAS mutation but that these mutations were not associated with TTR or OS (log-rank *p* = 0.425 for TTR; log-rank *p* = 0.137 for OS). In addition, several KRAS positive patients from a set of clinical trials did not respond to treatment with epidermal growth factor receptor inhibitors, cetuximab, or panitumumab (Artale et al., 2008; Van Cutsem et al., 2008) suggesting that these mutations may also be implicated in therapeutic response. Notably, the National Comprehensive Cancer Network guidelines suggest that every colorectal cancer patient, once confirmed to have developed liver metastases, should be screened for KRAS mutations. KRAS mutations serve as a predictor of unfavorable prognosis for colorectal cancer patients in both stage II and III tissues and indicate that these patients could benefit from postoperative FOLFOX chemotherapy (Deng et al., 2015). Given this more studies should investigate the underlying mechanisms of *KRAS* mutation-mediated effects on chemo- and immunotherapy.

We used GSEA analyses to compare the differences between G2 and G1 at the hallmark pathway level. Of the significantly enriched pathways, we found that coagulation, Myc-Target-V2, and epithelial-mesenchymal transition (EMT) were the most significantly correlated with the G2 group, which included patients with poorer outcomes than the G1 group. Most cancer patients demonstrate some biochemical evidence of the systematic activation of coagulation at diagnosis, and hemostatic changes may disappear after curative treatment. Iversen and Thorlacius-Ussing (2003) reported coagulation reactivation in response to cancer recurrence which was demonstrated by significantly increased plasma thrombin antithrombin III complex and Serum ferritin expression. Mandoj et al. (2018) established a risk predicting signature for OS which was shown to be closely associated with age (*p* = 0.043), tumor size (*p* = 0.001), levels of D-dimer (*p* = 0.029), and factor VIII (*p* = 0.087) when evaluated using a multivariate model. Coagulation abnormalities in cancer patients increase the tendency of these patients to develop both hemorrhages and thrombosis (Falanga et al., 2013) and evidence from basic research suggests that hemostatic components and cancer biology interact in multiple ways.

In summary, the PAS-based features and crosstalk evaluation provide an accurate and robust stratification of colorectal cancer patients. This stratification can be clearly linked to prognosis and the signature holds the promise of facilitating precision medicine for colorectal cancer patients.

## Data Availability

Publicly available datasets were analyzed in this study. This data can be found here: TCGA testing cohort, (B) GSE17537 cohort, (C) GSE29623 cohort, (D) GSE87211.
