# Supplementary material for: Molecular Subclassification Based on Crosstalk Analysis Improves Prediction of Prognosis in Colorectal Cancer
Source: Front Genet. 2021 Nov 4;12:689676. doi: 10.3389/fgene.2021.689676 (PMC8600263; doi:10.3389/fgene.2021.689676)
Supplement: Supplementary file 1 [file DataSheet2.PDF]

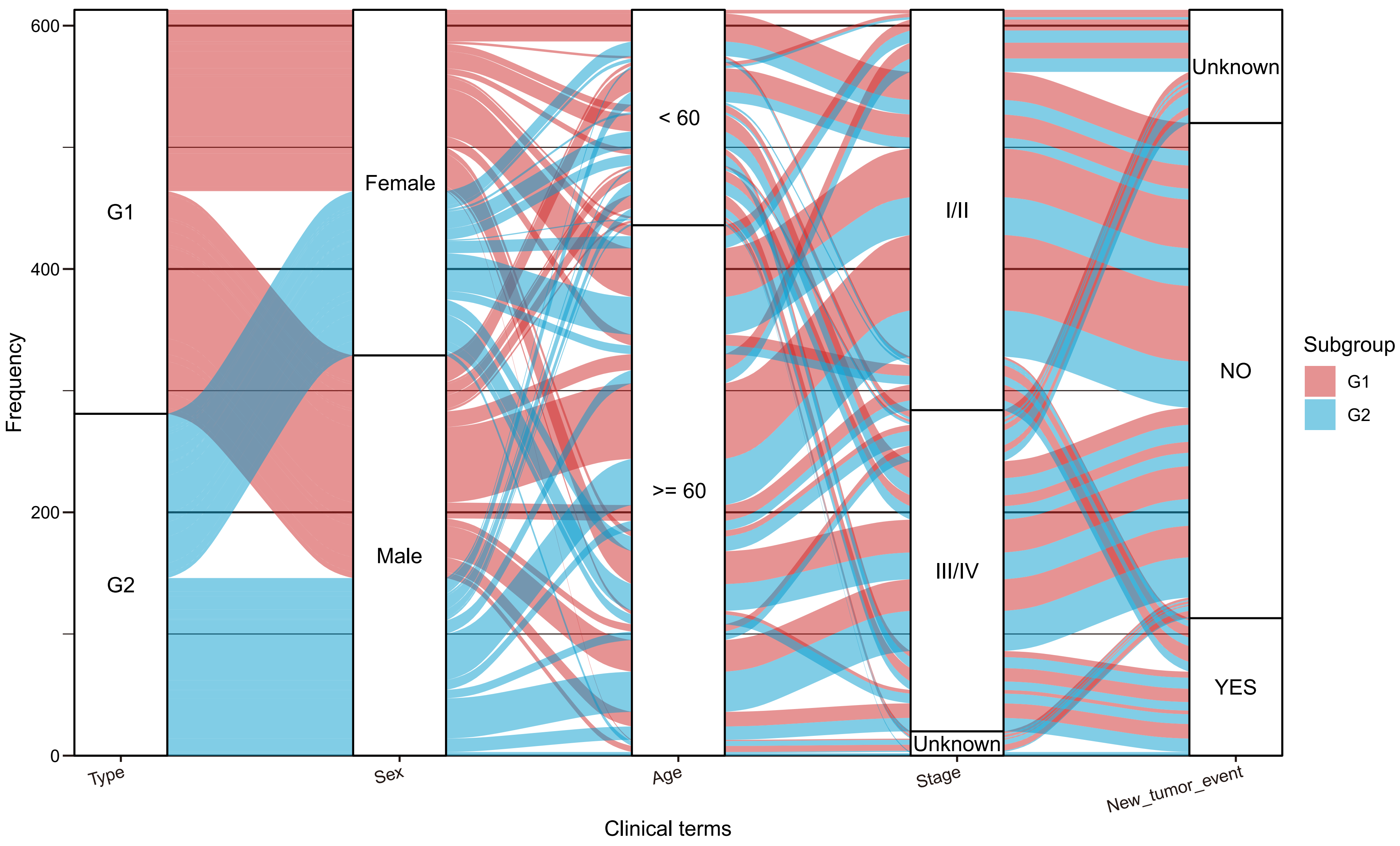

Fig. S2. Sankey diagram analyses. Sankey diagram analyses displayed the distributions of G2 and G1 subgroups in different clinicopathological subgroups, such as age, sex, clinical stage, and new tumor event. G2: aggressive (higher-risk survival) subtype; G1: moderate (lower-risk survival) subtype.
