# Supplementary material for: Molecular Subclassification Based on Crosstalk Analysis Improves Prediction of Prognosis in Colorectal Cancer
Source: Front Genet. 2021 Nov 4;12:689676. doi: 10.3389/fgene.2021.689676 (PMC8600263; doi:10.3389/fgene.2021.689676)
Supplement: Supplementary file 3 [file DataSheet4.PDF]

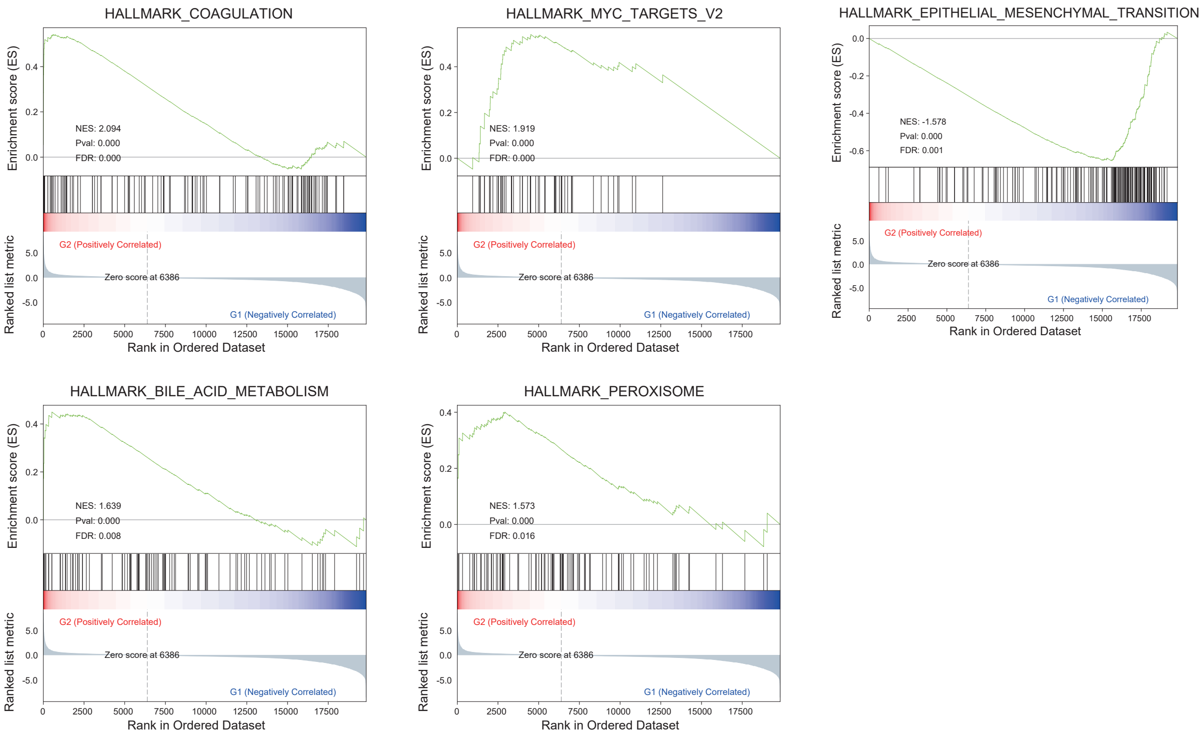

Fig. S4. Hallmark gene-set enrichment analysis (GSEA) analyses to identify the difference between G2 and G1 subgroups at the pathway level.
