# Supplementary material for: Molecular Subclassification Based on Crosstalk Analysis Improves Prediction of Prognosis in Colorectal Cancer
Source: Front Genet. 2021 Nov 4;12:689676. doi: 10.3389/fgene.2021.689676 (PMC8600263; doi:10.3389/fgene.2021.689676)
Supplement: Supplementary file 6 [file DataSheet3.PDF]

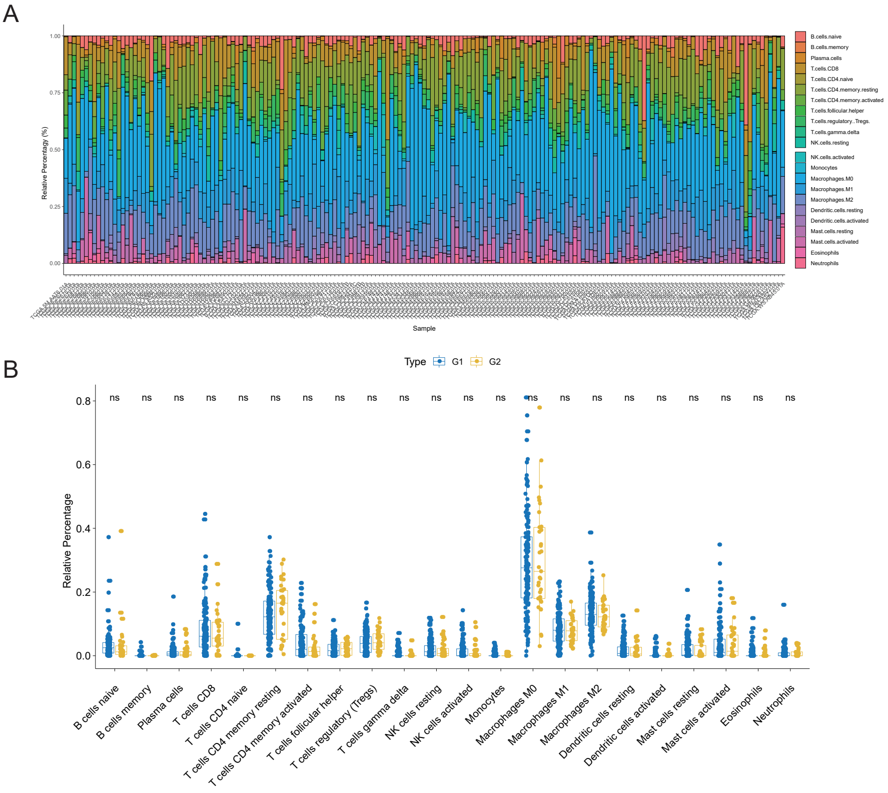

Fig. S3. CIBERSORT analyses displayed the infiltrated immunocyte differences between G2 and G1 subgroups. A. CIBERSORT analyses displayed the types of infiltrated immunocytes in each sample. B. Comparison between G2 and G1 subgroups at infiltrated immunocyte level.
