# Supplementary figures and images for: Molecular Subclassification Based on Crosstalk Analysis Improves Prediction of Prognosis in Colorectal Cancer
Source: Front Genet. 2021 Nov 4;12:689676. doi: 10.3389/fgene.2021.689676 (PMC8600263; doi:10.3389/fgene.2021.689676)

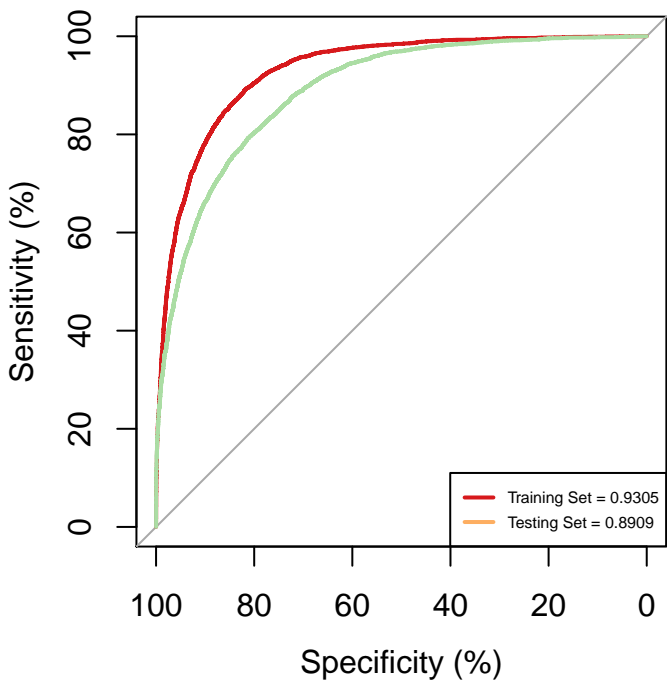

Fig. S1. The ROC curves of the training dataset and testing dataset.

Supplement: Supplementary file 8 [file DataSheet1.PDF]
